# Melioidosis in Patients with COVID-19 Exposed to Contaminated Tap Water, Thailand, 2021

**DOI:** 10.3201/eid3004.231476

**Published:** 2024-04

**Authors:** Panupong Tantirat, Yotsathon Chantarawichian, Pantila Taweewigyakarn, Somkid Kripattanapong, Charuttaporn Jitpeera, Pawinee Doungngern, Chadaporn Phiancharoen, Ratanaporn Tangwangvivat, Soawapak Hinjoy, Anupong Sujariyakul, Premjit Amornchai, Gumphol Wongsuvan, Viriya Hantakun, Vanaporn Wuthiekanun, Janjira Thaipadungpanit, Nicholas R. Thomson, David A.B. Dance, Claire Chewapreecha, Elizabeth M. Batty, Direk Limmathurotsakul

**Affiliations:** Saraburi Hospital, Mueang Saraburi District, Saraburi, Thailand (P. Tantirat);; Ministry of Public Health, Nonthaburi, Thailand (P. Tantirat, P. Taweewigyakarn, S. Kripattanapong, C. Jitpeera, P. Doungngern, C. Phiancharoen, R. Tangwangvivat, S. Hinjoy, A Sujariyakul);; Kaeng Khoi Hospital, Kaeng Khoi District, Saraburi (Y. Chantarawichian);; Mahidol University Mahidol-Oxford Tropical Medicine Research Unit, Bangkok, Thailand (P. Amornchai, G. Wongsuvan, V. Hantakun, V. Wuthiekanun, J. Thaipadungpanit, C. Chewapreecha, E.M. Batty, D. Limmathurotsakul);; Mahidol University, Bangkok (J. Thaipadungpanit, C. Chewapreecha, D. Limmathurotsakul);; Wellcome Sanger Institute, Hinxton, United Kingdom (N.R. Thomson, C. Chewapreecha);; Lao-Oxford-Mahosot Hospital-Wellcome Trust Research Unit, Vientiane, Lao People’s Democratic Republic (D.A.B. Dance);; London School of Hygiene and Tropical Medicine, London, UK (D.A.B Dance);; University of Oxford Centre for Tropical Medicine and Global Health, Oxford, UK (D.A.B. Dance, E.M. Batty, D. Limmathurotsakul)

**Keywords:** melioidosis, COVID-19, tap water, *Burkholderia pseudomallei*, coronavirus disease, SARS-CoV-2, severe acute respiratory syndrome coronavirus 2, viruses, respiratory infections, zoonoses, vaccine-preventable diseases, Thailand

## Abstract

In September 2021, a total of 25 patients diagnosed with COVID-19 developed acute melioidosis after (median 7 days) admission to a COVID-19 field hospital in Thailand. Eight nonpotable tap water samples and 6 soil samples were culture-positive for *Burkholderia pseudomallei*. Genomic analysis suggested contaminated tap water as the likely cause of illness.

Melioidosis is an infectious disease caused by the gram-negative bacillus *Burkholderia pseudomallei*, which is commonly present in soil and water in tropical countries (*1*,*2*). Naturally acquired infections result from skin inoculation, inhalation, or ingestion of *B. pseudomallei* (*1*). In 2020 and 2021, multiple COVID-19 field hospitals were set up in Thailand for cases of mild and moderate COVID-19 infection. We report 25 cases of acute melioidosis among patients diagnosed with COVID-19 who were being managed at a COVID-19 field hospital in Saraburi Province, central Thailand. Our study received ethical approval from the Committee of the Faculty of Tropical Medicine, Mahidol University (TMEC 24-006).

## The Study

On September 8, 2021, the Department of Disease Control, Ministry of Public Health, Thailand, was alerted to a cluster of 20 patients with culture-confirmed melioidosis (case nos. 1–20; Table). The 20 patients had been admitted to a single field hospital in Saraburi Province, which had been designated a treatment facility for COVID-19, and were transferred to Kaeng Khoi Hospital, Saraburi Province, because they developed fever or pneumonia. Previously, Saraburi Province had diagnosed ≈8–12 culture-confirmed melioidosis cases per year (*2*). The outbreak investigation team suspected that nonpotable tap water (NPTW) was the source of infection because there were no other apparent sources (Appendix). The initial response included the immediate transfer of patients with diabetes and those who had received steroid therapy for COVID-19 to other hospitals, followed by re-emphasizing to staff the recommended prevention strategies for melioidosis (*3*). The recommendations included avoiding direct exposure to soil and environmental water and drinking only boiled or bottled water.

**Table:** Clinical characteristics of 25 patients with COVID-19 who developed acute melioidosis, Saraburi Province, Thailand, 2021*

Case no.	Age, y/sex	Underlying diseases	Dates of COVID-19–positive PCR/field hospital admission/first melioidosis symptoms	Clinical manifestations of melioidosis	Cultures positive for *B. pseudomallei* (collection date)	Parenteral treatment for acute melioidosis (start date–stop date)	Oral treatment for melioidosis (start date)	Outcome (discharge or death date)
1†	56/F	DM, chronic hepatitis B	Aug 16/Aug 27/Sep 1	Fever, dyspnea, pneumonia‡	Blood (Sep 1)	MEM (Sep 5–7), CAZ (Sep 7–10), MEM (Sep 10–24)	TMP/SMX (Sep 24)	Survived (Sep 29)
2	66/M	IHD, VHD, CHF	Aug 22/Aug 26/Sep 1	Cough, fever, dyspnea, pneumonia‡	Blood (Sep 1)	None	NA	Died (Sep 1)
3†	52/F	DM	Aug 24/Aug 26/Sep 2	Fever, dyspnea, pneumonia‡	Blood (Sep 2)	MEM (Sep 5–6), CAZ (Sep 6–Oct 9), TMP/SMX (Sep 20–30)	TMP/SMX (Sep 30), changed to AMC (Oct 9)	Survived (Oct 9)
4	62/M	DM	Aug 20/Aug 29/Sep 2	Fever, diarrhea, pneumonia‡	Blood (Sep 2)	None	NA	Died (Sep 3)
5	44/F	DM, allergic rhinitis	Aug 20/Aug 26/Sep 2	Fever, hypotension, pneumonia‡	Blood (Sep 2)	CAZ (Sep 4–5)	NA	Died (Sep 5)
6	34/M	DM, obesity	Aug 17/Aug 26/Sep 1	Hypotension, pneumonia‡	Blood (Sep 2)	CAZ (Sep 4–19)	TMP/SMX (Sep 19)	Survived (Sep 20)
7	58/F	DM, HT, DLP	Aug 17/Aug 22/Sep 2	Cough, dyspnea, pneumonia‡	Blood (Sep 2)	CAZ (Sep 5–20)	TMP/SMX (Sep 20)	Survived (Sep 23)
8	56/M	HT, CKD, gout	Aug 16/Aug 26/Sep 1	Fever, diarrhea	Blood (Sep 2)	None	NA	Died (Sep 2)
9†	60/F	DM, HT, DLP	Aug 23/Aug 27/Sep 1	Dyspnea, pneumonia‡	Blood (Sep 3)	MEM (Sep 3–4)	NA	Died (Sep 4)
10	63/F	DM	Aug 22/Aug 28/Sep 2	Fever, pneumonia‡	Blood (Sep 2)	CAZ (Sep 4–18)	AMC (Sep 18)	Survived (Sep 22)
11	60/M	Gout	Aug 24/Aug 31/Sep 5	Fever	Blood (Sep 5)	CAZ (Sep 4–19)	TMP/SMX (Sep 19)	Survived (Sep 21)
12†	56/F	Rheumatoid arthritis	Aug 22/Aug 31/Sep 4	Fever, pneumonia‡	Blood (Sep 4)	MEM (Sep 5–8), CAZ (Sep 8–19)	TMP/SMX (Sep 19)	Survived (Sep 20)
13†	36/F	DM, obesity	Aug 18/Aug 29/Sept 10	Dyspnea, pneumonia‡	Blood (Sep 6)	CAZ (Sep 8–23)	TMP/SMX (Sep 22)	Survived (Sep 24)
14	56/M	None	Aug 17/Aug 24/Sep 4	Fever, pneumonia‡	Blood (Sep 4)	MEM (Sep 5–8)	NA	Died (Sep 8)
15	59/F	HT	Aug 21/Sept 2/Sep 6	Dyspnea, pneumonia‡	Blood (Sep 6)	CAZ (Sep 6–20)	TMP/SMX (Sep 21)	Survived (Sep 23)
16	61/F	DM, HT	Aug 19/Aug 24/Sep 2	Dyspnea, pneumonia‡	Sputum (Sep 3)	CAZ (Sep 5–19)	TMP/SMX (Sep 19)	Survived (Sep 20)
17	73/M	COPD	Aug 23/Aug 25/Sep 3	Dyspnea, pneumonia‡	Blood (Sep 3)	CAZ (Sep 4–18)	AMC (Sep 18)	Survived (Sep 20)
18	66/M	DM, CKD, HT, DLP	Aug 23/Aug 30/Sep 3	Diarrhea, pneumonia‡	Blood (Sep 3)	CAZ (Sep 4–19)	TMP/SMX (Sep 19)	Survived (Sep 20)
19	62/M	DM, HT, DLP	Aug 24/Aug 27/Sep 2	Fever, dyspnea, pneumonia‡	Blood (Sep 3)	CAZ (Sep 4)	NA	Died (Sep 4)
20	60/F	DM, HT, DLP	Aug 20/Aug 27/Sep 3	Dyspnea, pneumonia‡	Blood (Sep 3)	Meropenems (Sep 3)	NA	Died (Sep 4)
21	36/M	DM, obesity	Aug 24/Aug 28/Sep 5	Fever, pneumonia‡	Blood (Sep 5)	CAZ (Sep 5–18)	TMP/SMX (Sep 19)	Survived (Sep 23)
22†	60/F	None	Aug 20/Aug 27/Sep 5	Dyspnea, pneumonia‡	Blood (Sep 10)	CAZ (Sep 5–19), MEM (Sep 19–Oct 13), TMP/SMX (Sep 22–Oct 12)	TMP/SMX (Oct 13)	Survived (Oct 19)
23	70/M	None	Aug 29/Aug 31/Sep 11	Fever Dizziness	Blood (Sep 11)	CAZ (Sep 11–25)	TMP/SMX (Sep 25)	Survived (Sep 26)
24†	52	None	Aug 18/Aug 22/Sep 11	Fever, dyspnea, pneumonia‡	Blood (Sep 13)	MEM (Sep 13–20), CAZ (Sep 20–Oct 9), TMP/SMX (Sep 20–Oct 9)	TMP/SMX (Oct 9)	Survived (Oct 9)
25	58	DM	Aug 29/Sep 2/Sept 10	Fever, pneumonia‡	Blood (Sep 14)	CAZ (14–28 Sep)	TMP-SMX (from Sep 28)	Survived (Sep 29)

*The discharge date was the date of discharge from hospital after completion of intensive phase treatment for melioidosis. For patients who died of acute melioidosis, the date of death is given. CKD, chronic kidney disease; DLP, dyslipidemia; DM, diabetes mellitus; HT, hypertension; CAZ, ceftazidime; IMP, imipenem; MEM, meropenem; AMC, amoxicillin/clavulanic acid; TMP/SMX, trimethoprim/sulfamethoxazole; NA, not applicable. †Transferred to Saraburi Hospital, Thailand. ‡Pneumonia was defined as the presence of a new or progressive radiographic infiltrate and >1 of 3 clinical features (fever >38°C, leukocytosis or leukopenia, and purulent secretions) suspected to be caused by acute melioidosis.

Health officials immediately planned and conducted an environmental investigation. During September 10–16, 2021, the outbreak investigation team collected samples from the field hospital, including 8 commercially bottled drinking water (CBDW) samples (500 mL–1 L), 37 NPTW samples from 10 locations (1-L samples; 3–4 samples per location at different time points), and 50 soil samples (100 g per sample) (Appendix). We isolated *B. pseudomallei* from the environmental samples according to previously described methods (*4**,**5*). None of the CBDW samples, 6 (12%) of 50 soil samples, and 8 (22%) of 37 NPTW samples (from 4 locations) were culture-positive for *B. pseudomallei*. The median quantitative count of *B. pseudomallei* in NPTW was 24.5 CFU/L (range 4–58 CFU/L) and in soil was 82 CFU/g (range <1–119 CFU/g). The outbreak investigation team found that the chlorination system for NPTW was not well maintained. Patients reported that they drank only CBDW and never drank NPTW, which was used for other domestic purposes, such as brushing their teeth, rinsing their mouths, and showering. The chlorination system was successfully repaired, and chlorine levels were maintained >1 ppm beginning on September 10. A further 5 melioidosis cases were identified, all of whom had been admitted before September 10. No new melioidosis cases among those who had stayed at the field hospital were reported after September 16.

Of the 25 patients diagnosed with melioidosis (Table), 12 (48%) were female and 13 (51%) male; median age was 59 (interquartile range 56–62, range 34–73) years. All patients had received a diagnosis of COVID-19, confirmed by PCR during August 16–29, 2021, and had been admitted to the field hospital in August 22–September 2, 2021. A total of 15 (60%) patients had diabetes, and all 25 (100%) patients had received steroids as part of their COVID-19 treatment. The date range of onset of symptoms attributed to melioidosis was September 1–11. The median time from admission to the field hospital to the onset of the melioidosis symptoms was 7 (interquartile range 5–9, range 4–20) days.

The most common clinical manifestations of melioidosis among patients in this cluster were secondary bacterial pneumonia (n = 22 patients [88%]) and fever (n = 15 [60%]) (Table). Clinical specimens that were culture positive for *B. pseudomallei* were blood (n = 24 [96%]) and sputum (n = 1 [4%]). In-hospital mortality for patients we studied was 32% (8/25). Of the fatal cases, 3 patients (case nos. 2, 4, and 8) died without receiving ceftazidime or meropenem, which are recommended parenteral antibiotics for treatment of melioidosis. A total of 17 (68%) cases completed >10 days of parenteral ceftazidime or meropenem and subsequently received a course of oral eradicative treatment.

We confirmed the first 20 clinical isolates and all environmental isolates as *B. pseudomallei* at the Mahidol-Oxford Tropical Medicine Research Unit laboratory, Bangkok, by using a combination of colony morphology on Ashdown agar, latex agglutination test, arabinose assimilation test, and antimicrobial susceptibility tests. Testing revealed that all clinical and environmental isolates were susceptible to ceftazidime, meropenem, and trimethoprim/sulfamethoxazole. We performed whole-genome sequencing on 19 clinical isolates, 8 NPTW isolates, and 6 soil isolates (1 colony per patient or sample). We excluded 1 clinical isolate from analysis because of low sequencing depth of 7.5×. We deposited sequences in the European Nucleotide Archive (https://www.ebi.ac.uk/ena) (accession numbers in Appendix Table). We mapped isolates to the K96243 reference genome and used variant calls to construct a phylogeny after masking recombinant fragments, repetitive regions, and known *B. pseudomallei* genomic islands (*6*). We used genome assemblies to call multilocus sequence types (STs).

We categorized the isolates by both phylogenetic and multilocus sequence typing, and they clustered consistently into 4 groups. The largest cluster was ST689 and included all 19 blood and sputum samples, as well as 6 of 8 NPTW samples (Figure). The remaining soil and NPTW isolates formed 3 separate clusters (ST107, ST303, and ST315). Within the ST689 cluster, the isolates were closely related but not identical (12–98 SNP differences between isolates). The NPTW isolates were interspersed with clinical isolates in this cluster, suggesting that contaminated NPTW was a possible source of infection for these patients. Dating analysis was not feasible because of the absence of clock signals in the phylogeny.

**Figure:**
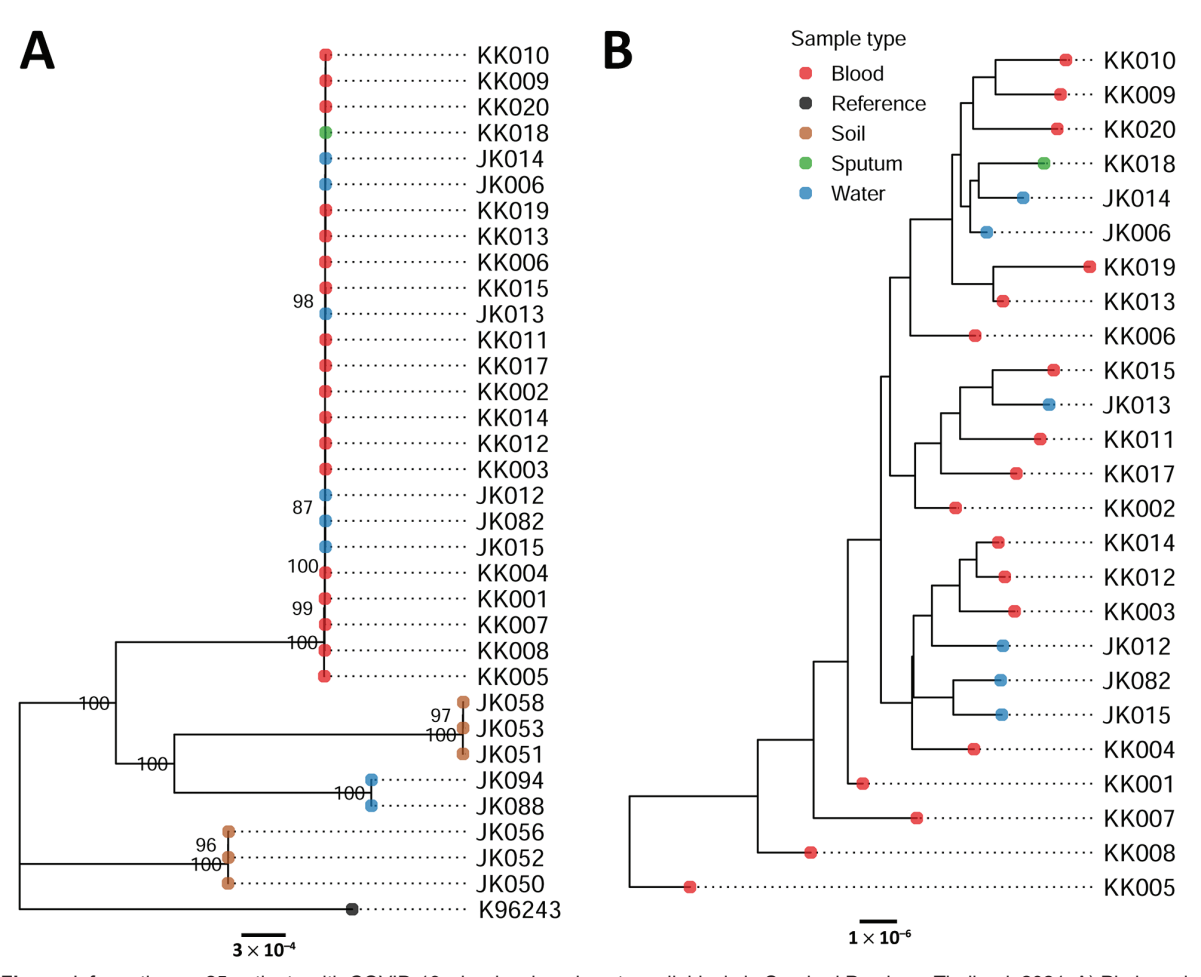
Information on 25 patients with COVID-19 who developed acute melioidosis in Saraburi Province, Thailand, 2021. A) Phylogenic tree of 19 clinical isolates and 14 environmental isolates. The K96243 reference strain is used to root the tree. B) Tree showing only the clinical and environmental isolates within the ST689 cluster. Scale bar shows number of nucleotide differences.

## Conclusions

Our study highlights that patients with viral infections (e.g., COVID-19) may be at risk for infection and death caused by melioidosis if exposed to NPTW contaminated by *B. pseudomallei*. Diabetes mellitus and conditions that impair innate and adaptive immune responses, particularly steroid use, are important risk factors for melioidosis (*1*). Diabetes mellitus is also a risk factor for COVID-19, and steroid treatment is recommended for patients with COVID-19 pneumonia (*7*). Therefore, unsurprisingly, co-infections with COVID-19 and *B. pseudomallei* have been reported occasionally (*8*,*9*; D. Chit Yee et al., unpub. data, https://wellcomeopenresearch.org/articles/7-160), including 1 of the 4 patients detected during the multistate outbreak of melioidosis caused by an imported aromatherapy spray in the United States (*10*), and now this cluster. Previous reports of co-infection with influenza A (*11*,*12*) or COVID-19 (*9*) and *B. pseudomallei* suggested that melioidosis could be reactivated from a latent focus following viral infection. However, the timeline of the cluster, the identified source, and genomic analysis suggest that the patients in this cluster represented recently acquired secondary infections after COVID-19. The route of infection in this cluster was probably skin exposure to contaminated NPTW at a high-infecting dose, although ingestion or inhalation are also possible. 

An unknown proportion of melioidosis patients in melioidosis-endemic areas could be related to exposure to contaminated NPTW. More studies on the effects of *B. pseudomallei*-contaminated NPTW and its disinfection (*13*) in melioidosis endemic areas are required. Because general recommendations for melioidosis prevention (*3*) do not emphasize the disinfection of NPTW, those recommendations may be inadequate and should be revisited.

Appendix More information for melioidosis in patients with COVID-19 exposed to contaminated tap water, Thailand, 2021.
