# Supplementary material for: Melioidosis in Patients with COVID-19 Exposed to Contaminated Tap Water, Thailand, 2021
Source: Emerg Infect Dis. 2024 Apr;30(4):791–4. doi: 10.3201/eid3004.231476 (PMC10977828; doi:10.3201/eid3004.231476)
Supplement: Appendix — More information for melioidosis in patients with COVID-19 exposed to contaminated tap water, Thailand, 2021. [file 23-1476-Techapp-s1.pdf]

Article DOI: http://doi.org/10.3201/eid3004.231476

EID cannot ensure accessibility for supplementary materials supplied by authors. Readers who have difficulty accessing supplementary content should contact the authors for assistance.

Melioidosis in Patients with COVID-19 Exposed to Contaminated Tap Water, Thailand, 2021

Appendix

Our study received ethical approval from the Committee of the Faculty of Tropical Medicine, Mahidol University (TMEC 24–006). Individual consent was not sought from the patients as this was a retrospective study, and the Ethical and Scientific Review Committees approved the process.

On 8th September 2021, the Department of Disease Control, Ministry of Public Health, Thailand was alerted to a cluster of 20 patients with culture-confirmed melioidosis. Using initial information obtained from the field hospital, Kaeng Khoi Hospital and Saraburi Hospital, the outbreak investigation team suspected that nonpotable tap water (NPTW) was the most likely source of infection. This was because (a) all COVID-19 patients in the cluster had been quarantined in five buildings in the field hospital, all buildings had concrete floors, they had not been exposed to soil, rice fields or surface water before the onset of symptoms, and, therefore, skin exposure to environmental soil or water was not primarily suspected as the route of infection; (b) there had been no storms or heavy rainfall before the onset of symptoms, and, therefore, inhalation of environmental aerosols was not primarily suspected as the route of infection; (c) all COVID-19 patients in the field hospital drank commercial bottled drinking water (CBDW) which was donated to the field hospital, CBDW was also donated to other field hospitals but there were no melioidosis cases from those field hospitals, and, therefore, CBDW was not primarily suspected as the source of infection; (d) food was cooked and prepared for the field hospital according to hospital standards; therefore, food was not primarily suspected as the source of infection; and (e) all patients exposed to NPTW for their daily activities such as brushing their teeth, rinsing their mouths and showering, there were no records of quality testing of NPTW at the field hospital, and, consequently, NPTW was suspected as the most likely source

of infection. Patients reported that they drank only CBDW and never drank NPTW. There were shower facilities in the field hospital that used NPTW, but there were no bathtubs.

The initial response included transferring diabetic patients and those who had received steroid therapy for COVID-19 to other hospitals immediately. This was because diabetes and steroid use are known as important risk factors for melioidosis in Thailand. A case-control study in 1999 in Thailand found that the use of herbal medicine, which might contain steroids, was more common in melioidosis patients that that in patients with non-infectious diseases (10% versus 5%) (*1*). Steroid use was also observed as a risk factor in several melioidosis case reports (*2*). A case-control study in 2013 in Thailand found that steroid intake was independently associated with melioidosis (adjusted conditional odds ratio 3.1; 95% confidence interval 1.4–6.9, p = 0.006) (*3*).

An environmental investigation was immediately planned and conducted. On 10th September 2021, 50 soil samples (1 sample/sampling point; 100 g/sample) were collected. Six sampling points were around building A, six sampling points around building B, six sampling points around building C, six sampling points around building D, 10 sampling points around building E, four sampling points around building F, seven sampling points around building I, two sampling points around a building that was set up for chest radiography, two sampling points around the parking area, and one sampling point in front of building Z. We anonymized the names of the buildings. All COVID-19 patients stayed in buildings A to E. On 10th September, eight CBDW and 17 NPTW samples (one liter/sample) were also collected. The 17 NPTW samples were collected from taps in buildings A, B, C, D, E, F, I and another three tap locations within the field hospital. NPTW samples were collected at noon and in the evening from each tap. The evening samples were not obtained from the taps in buildings B, C and D. On 13th September, another 10 NPTW samples were collected from each tap. On 16th September, another 10 NPTW samples were collected from each tap.

None of the CBDW samples, six of 50 soil samples (12%) and eight of 37 NPTW samples (22%) (from four locations) were culture positive for *B. pseudomallei*. The six soil samples culture positive for *B. pseudomallei* were collected from six sampling points around building E. The eight NPTW samples culture positive for *B. pseudomallei* were collected from

the tap in building E (four samples), the tap in building F (three samples) and the tap in building I (one sample).

Of the eight fatal cases, three died without receiving ceftazidime or meropenem, which are recommended parenteral antibiotics for treatment of melioidosis (Case Nos. 2, 4 and 8). Those three cases died before the results of blood cultures were available. For example, Case No. Two had blood specimens collected for bacterial culture on 1st September and died on the same day. Case No. Eight had blood specimens collected for culture on 2nd September and died on the same day. In Thailand, it is not uncommon that melioidosis patients die before the results of bacterial culture are available (*4*). The median time between taking the blood culture and the sample flagging as positive was 1 day (interquartile range 1–2 days) (*5*,*6*), and it would take another 1 or 2 calendar days for microbiology laboratories to subculture and confirm that the isolate was *B. pseudomallei*. Therefore, ceftazidime or meropenem are recommended for empirical treatment for community-acquired sepsis patients in melioidosis-endemic areas (*7*). Nonetheless, Saraburi province is in central Thailand and the incidence of melioidosis is relatively low compared to that in northeast Thailand (*8*). Therefore, clinicians did not suspect melioidosis during the very early phase of the cluster (i.e., 1st-3rd September). After the confirmation of the bacterial culture results of the first few cases, ceftazidime or meropenem were then used for empirical treatment among COVID-19 patients who were transferred from the field hospital with acute sepsis or pneumonia.

The route of infection in this cluster was probably skin exposure to contaminated NPTW at a high infecting dose, although ingestion or inhalation are also possible. In this cluster, patients had a high proportion of bacteraemia, pneumonia, and mortality. This could be associated with underlying COVID-19 and diabetes, immunosuppression caused by the steroid therapy, and other confounding factors. Diarrhea was noted as one of the clinical presentations in Cases 4, 8 and 18. This could be because (a) contaminated NPTW was ingested while rinsing mouths or showering and gastrointestinal tract infection was primarily involved, or (b) *B. pseudomallei* was disseminated to the gastrointestinal tract after skin exposure to contaminated NPTW.

To our knowledge, this is the largest cluster of *B. pseudomallei* in humans acquired from contaminated NPTW. Acquisition of *B. pseudomallei* infection in humans from contaminated NPTW has previously been reported from Australia (*9–11*) and Thailand (*12*,*13*). A study

previously reported 159 infected swine from 1981 to 1983 in southern Queensland (*14*), where the water supply was suspected as the source of infection although this could not be confirmed.

The study has some limitations. First, the study does not have additional detailed information, such as individual levels of exposure to NPTW, COVID-19 presentations and symptoms and dosages of steroid use. According to the COVID-19 guidelines in Thailand, none of the COVID-19 patients admitted or transferred to the field hospital had symptoms of severe COVID-19, such as high fever (≥39°C), requirement for oxygen supplementation, severe co-morbidities, or other conditions that attending physicians considered to necessitate hospital admission. Second, the study team does not have information on other COVID-19 patients who were admitted to the field hospital and did not develop acute melioidosis. Therefore, we could not evaluate odds ratios or hazard ratios for the risk of acquiring melioidosis.

References

1. Suputtamongkol Y, Chaowagul W, Chetchotisakd P, Lertpatanasuwun N, Intaranongpai S, Ruchutrakool T, et al. Risk factors for melioidosis and bacteremic melioidosis. Clin Infect Dis. 1999;29:408–13. PubMed https://doi.org/10.1086/520223

2. Cheng AC, Currie BJ. Melioidosis: epidemiology, pathophysiology, and management. Clin Microbiol Rev. 2005;18:383–416. PubMed https://doi.org/10.1128/CMR.18.2.383-416.2005

3. Limmathurotsakul D, Kanoksil M, Wuthiekanun V, Kitphati R, deStavola B, Day NP, et al. Activities of daily living associated with acquisition of melioidosis in northeast Thailand: a matched case-control study. PLoS Negl Trop Dis. 2013;7:e2072. PubMed https://doi.org/10.1371/journal.pntd.0002072

4. Chantratita N, Phunpang R, Yarasai A, Dulsuk A, Yimthin T, Onofrey LA, et al. Characteristics and One Year Outcomes of Melioidosis Patients in Northeastern Thailand: A Prospective, Multicenter Cohort Study. Lancet Reg Health Southeast Asia. 2023;9:9. PubMed https://doi.org/10.1016/j.lansea.2022.100118

5. Teerawattanasook N, Limmathurotsakul D, Day NPJ, Wuthiekanun V. Short report: Failure of *Burkholderia pseudomallei* to grow in an automated blood culture system. Am J Trop Med Hyg. 2014;91:1173–5. PubMed https://doi.org/10.4269/ajtmh.14-0018

6. Chantratita N, Tandhavanant S, Wongsuvan G, Wuthiekanun V, Teerawattanasook N, Day NPJ, et al. Rapid detection of *Burkholderia pseudomallei* in blood cultures using a monoclonal antibody-based immunofluorescent assay. Am J Trop Med Hyg. 2013;89:971–2. PubMed https://doi.org/10.4269/ajtmh.13-0212

7. Meumann EM, Limmathurotsakul D, Dunachie SJ, Wiersinga WJ, Currie BJ. *Burkholderia pseudomallei* and melioidosis. Nat Rev Microbiol. 2023. PubMed

8. Hantrakun V, Kongyu S, Klaytong P, Rongsumlee S, Day NPJ, Peacock SJ, et al. Clinical Epidemiology of 7126 Melioidosis Patients in Thailand and the Implications for a National Notifiable Diseases Surveillance System. Open Forum Infect Dis. 2019;6:ofz498. PubMed https://doi.org/10.1093/ofid/ofz498

9. McRobb E, Sarovich DS, Price EP, Kaestli M, Mayo M, Keim P, et al. Tracing melioidosis back to the source: using whole-genome sequencing to investigate an outbreak originating from a contaminated domestic water supply. J Clin Microbiol. 2015;53:1144–8. PubMed https://doi.org/10.1128/JCM.03453-14

10. Inglis TJ, Garrow SC, Henderson M, Clair A, Sampson J, O'Reilly L, et al. *Burkholderia pseudomallei* traced to water treatment plant in Australia. Emerg Infect Dis. 2000;6:56–9. PubMed

11. Currie BJ, Mayo M, Anstey NM, Donohoe P, Haase A, Kemp DJ. A cluster of melioidosis cases from an endemic region is clonal and is linked to the water supply using molecular typing of *Burkholderia pseudomallei* isolates. Am J Trop Med Hyg. 2001;65:177–9. PubMed https://doi.org/10.4269/ajtmh.2001.65.177

12. Limmathurotsakul D, Wongsuvan G, Aanensen D, Ngamwilai S, Saiprom N, Rongkard P, et al. Melioidosis caused by *Burkholderia pseudomallei* in drinking water, Thailand, 2012. Emerg Infect Dis. 2014;20:265–8. PubMed https://doi.org/10.3201/eid2002.121891

13. Thaipadungpanit J, Chierakul W, Pattanaporkrattana W, Phoodaeng A, Wongsuvan G, Huntrakun V, et al. *Burkholderia pseudomallei* in water supplies, southern Thailand. Emerg Infect Dis. 2014;20:1947–9. PubMed https://doi.org/10.3201/eid2011.140832

14. Ketterer PJ, Webster WR, Shield J, Arthur RJ, Blackall PJ, Thomas AD. Melioidosis in intensive piggeries in south eastern Queensland. Aust Vet J. 1986;63:146–9. PubMed https://doi.org/10.1111/j.1751-0813.1986.tb02953.x

Appendix Table. Accession numbers of 18 blood isolates, 1 sputum isolate, 8 water isolates, and 6 soil isolates of *Burkholderia pseudomallei* sequenced in this study

Sample ID	SRA accession
JK052	ERS14632051
JK053	ERS14632052
JK058	ERS14632053
JK094	ERS14632057
JK006	ERS14632043
JK012	ERS14632045
JK013	ERS14632046
JK014	ERS14632049
JK015	ERS14632047
JK050	ERS14632050
JK051	ERS14632048
JK056	ERS14632054
JK082	ERS14632055
JK088	ERS14632056
KK001	ERS14632058
KK002	ERS14632059
KK003	ERS14632060
KK004	ERS14632061
KK005	ERS14632062
KK006	ERS14632063
KK007	ERS14632064
KK008	ERS14632066
KK009	ERS14632065
KK010	ERS14632067
KK011	ERS14632068
KK012	ERS14632070
KK013	ERS14632069
KK014	ERS14632072
KK015	ERS14632071
KK016	ERS14632073
KK017	ERS14632077
KK018	ERS14632076
KK019	ERS14632075
KK020	ERS14632074
